# Mechanistic analysis and significance of sphingomyelinase‐mediated decreases in transepithelial CFTR currents in nHBEs

**DOI:** 10.14814/phy2.15023

**Published:** 2021-09-13

**Authors:** Kirsten A. Cottrill, Vincent D. Giacalone, Camilla Margaroli, Robert J. Bridges, Michael Koval, Rabindra Tirouvanziam, Nael A. McCarty

**Affiliations:** ^1^ Molecular and Systems Pharmacology PhD Program Emory University Atlanta Georgia USA; ^2^ Immunology and Molecular Pathogenesis PhD Program Emory University Atlanta Georgia USA; ^3^ Department of Medicine Division of Pulmonary Allergy & Critical Care Medicine University of Alabama at Birmingham Birmingham Alabama USA; ^4^ Program in Protease/Matrix Biology University of Alabama at Birmingham Birmingham Alabama USA; ^5^ Department of Physiology and Biophysics Center for Genetic Diseases Chicago Medical School North Chicago Illinois USA; ^6^ Department of Medicine Division of Pulmonary, Allergy, Critical Care and Sleep Medicine and Department of Cell Biology Emory University Atlanta Georgia USA; ^7^ Department of Pediatrics and Children’s Healthcare of Atlanta Center for Cystic Fibrosis and Airways Disease Research Emory University School of Medicine Atlanta Georgia USA

**Keywords:** AMPK, cystic fibrosis transmembrane conductance regulator, epithelial cells, modulator therapy, neutrophils, sphingomyelinase

## Abstract

Loss of function of the cystic fibrosis transmembrane conductance regulator (CFTR) causes cystic fibrosis (CF). In the lungs, this manifests as immune cell infiltration and bacterial infections, leading to tissue destruction. Previous work has determined that acute bacterial sphingomyelinase (SMase) decreases CFTR function in bronchial epithelial cells from individuals without CF (nHBEs) and with CF (cfHBEs, homozygous ΔF508‐CFTR mutation). This study focuses on exploring the mechanisms underlying this effect. SMase increased the abundance of dihydroceramides, a result mimicked by blockade of ceramidase enzyme using ceranib‐1, which also decreased CFTR function. The SMase‐mediated inhibitory mechanism did not involve the reduction of cellular CFTR abundance or removal of CFTR from the apical surface, nor did it involve the activation of 5′ adenosine monophosphate‐activated protein kinase. In order to determine the pathological relevance of these sphingolipid imbalances, we evaluated the sphingolipid profiles of cfHBEs and cfHNEs (nasal) as compared to non‐CF controls. Sphingomyelins, ceramides, and dihydroceramides were largely increased in CF cells. Correction of ΔF508‐CFTR trafficking with VX445 + VX661 decreased some sphingomyelins and all ceramides, but exacerbated increases in dihydroceramides. Additional treatment with the CFTR potentiator VX770 did not affect these changes, suggesting rescue of misfolded CFTR was sufficient. We furthermore determined that cfHBEs express more acid‐SMase protein than nHBEs. Lastly, we determined that airway‐like neutrophils, which are increased in the CF lung, secrete acid‐SMase. Identifying the mechanism of SMase‐mediated inhibition of CFTR will be important, given the imbalance of sphingolipids in CF cells and the secretion of acid‐SMase from cell types relevant to CF.

## 1 INTRODUCTION

Cystic fibrosis (CF) is caused by loss of function of the cystic fibrosis transmembrane conductance regulator (CFTR), an apically located chloride and bicarbonate ion channel in airway epithelial cells (Riordan et al., 1989; Rommens et al., 1989). CFTR is activated by protein kinase A (PKA)‐mediated phosphorylation of its regulatory (R) domain, and is gated by the binding and hydrolysis of ATP (Berger et al., 1991; Frizzell & Hanrahan, 2012). Currently, most people living with CF succumb to pulmonary failure caused in part by chronic airway obstruction, immune cell infiltration, and lung infections (Cystic Fibrosis Foundation, 2018; Elborn, 2016).

Fortunately, highly effective modulator therapies have been developed recently that target the primary CFTR defect (Guimbellot et al., 2017; Joshi et al., 2019). However, we have shown that these modulator therapies are unable to recover a loss of CFTR currents and CFTR‐mediated apical membrane conductance following exposure to sphingomyelinase (SMase), a bacterial virulence factor (Cottrill et al., 2021; Stauffer et al., 2017). SMase converts sphingomyelin into ceramide and phosphocholine. Sphingolipids are well known to be involved in CF inflammatory lung disease, as has been detailed elsewhere (Maceyka & Spiegel, 2014). Harmful stimuli such as oxidative stress, tumor necrosis factor α, and lipopolysaccharide, all associated with the CF lung (Dua et al., 2019; Vliet et al., 2018), can increase the activity of endogenous neutral‐SMase as well as the secretion and activity of endogenous acid‐SMase in lung epithelial cells (Chan & Goldkorn, 2000; Jenkins et al., 2010; Kornhuber et al., 2015; Lavrentiadou et al., 2001). Furthermore, both *Pseudomonas aeruginosa* and *Staphylococcus aureus*, the two bacteria most commonly found in CF airways (Cystic Fibrosis Foundation, 2018), secrete enzymes with SMase activity (Barker et al., 2004; Huseby et al., 2007).

To add to the relevance of this question, much work has been performed recently that identifies sphingolipid imbalances in CF bronchial epithelial cells compared to non‐CF controls. Dihydroceramides increase under hypoxic conditions and are increased in bronchial epithelial cell models of CF (Devlin et al., 2011; Hamai et al., 2009). Furthermore, multiple groups have found that ceramides are increased in polarized primary bronchial epithelial cells from people living with CF (cfHBEs, homozygous or heterozygous for the ΔF508‐CFTR mutation), as compared to cells from non‐CF subjects (nHBEs) (Gardner et al., 2020; Loberto et al., 2020). Importantly, however, conflicting data also has been found in which overall ceramide levels are actually decreased in CF cells compared to non‐CF cells. In this instance, it also was found that the ratio of long‐chain ceramides to very‐long‐chain ceramides is increased (Veltman et al., 2021). The reason for this discrepancy in results between labs can only be speculated upon at this time.

**FIGURE 1:**
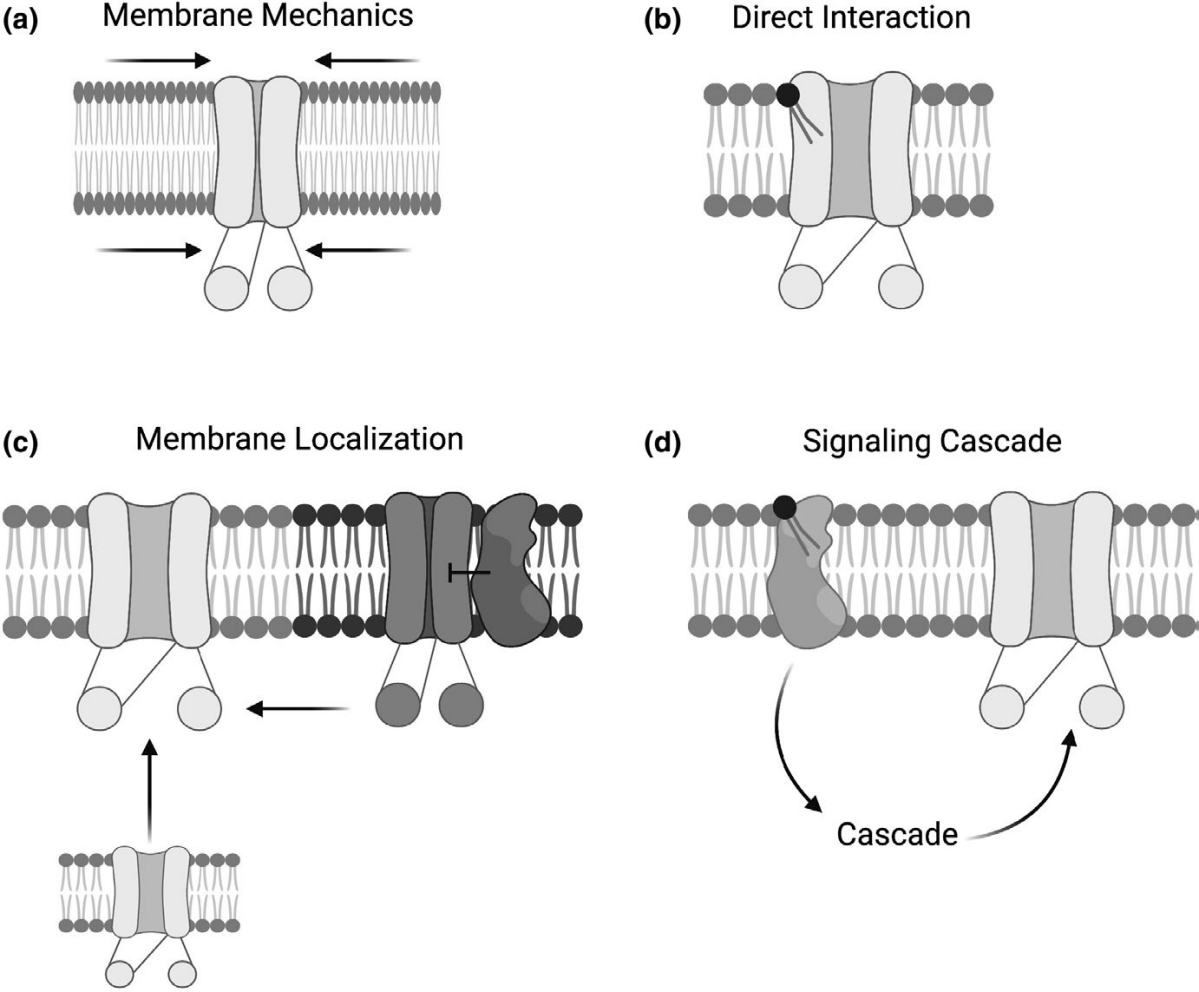
Schematic of how lipids can affect channel activity, including (a) mechanical properties of the membrane, such as its stiffness, (b) direct interactions between lipids and the channel, (c) the localization of the channel either within the cell or the plasma membrane, and (d) initiation of a signaling cascade that affects channel activity. This figure was made using BioRender

Given the relevance of these sphingolipid imbalances and their modulating enzymes, more work is needed to understand the mechanism of SMase‐mediated inhibition of transepithelial CFTR currents. Decreased transepithelial anion current after SMase treatment could be due to the loss of sphingomyelin, the production of phosphocholine or ceramide, the production of ceramide derivatives such as sphingosine‐1‐phosphate, some combination of these, or some other effect of SMase. Furthermore, lipids have the potential to affect membrane channel activity by a number of means (Figure 1) namely: (a) modulating membrane mechanics, (b) directly interacting with the channel and affecting its structure/function, (c) changing surface localization of the channel (either total surface localization or microdomain localization), and (d) initiating signaling cascades that result in modifications to the channel (Cottrill et al., 2020).

One of the goals of the work presented here was to understand in more detail the mechanism by which SMase decreased transepithelial anion currents in primary airway epithelial cells. Another goal was to determine the sphingolipid imbalance in CF airway epithelial cells as compared to non‐CF control cells, given the conflicting current literature discussed above. Furthermore, we were interested in the effects of relevant CFTR modulators on this sphingolipid imbalance, because again conflicting literature exists (Liessi et al., 2020; Veltman et al., 2021). Lastly, we sought to determine the relevance of acid‐SMase secretion from bronchial epithelial cells, as well as from airway‐like neutrophils, which are highly prevalent in the CF lung (Giacalone et al., 2020).

## 2 MATERIALS AND METHODS

Unless otherwise specified, chemical reagents were purchased from MilliporeSigma.

### 2.1 Airway epithelial cells from human donors

Two different methods for preparing and culturing primary airway epithelial cells were used, as described previously (Cottrill et al., 2021). One method is based on that developed in the Randell laboratory (Fulcher et al., 2005). Briefly, primary human bronchial (HBE) cells were isolated from donor lung explants under an Institutional Review Board‐approved protocol through the CF@LANTA Experimental Models Core, and nasal (HNE) cells were isolated by nasal scraping. Cells were plated on Costar 3470 Transwell plates (0.4 μm pore size, polyester, Corning), were transitioned to air–liquid interface (ALI) after 2 days, and were maintained in ALI medium for at least 3 weeks. This ALI medium was based on previous formulations (Fulcher et al., 2005), with modifications to glucose (150 mg/dl; 8.3 mM), CaCl_2_ (1 mM), heparin (2 µg/ml), l‐glutamine (2.5 mM), hydrocortisone (960 mg/ml), bovine pituitary extract (20 µg/ml), and Mg^2+^ (0.5 µM). For CF cells (cfHBEs, cfHNEs), which were homozygous for the ΔF508‐CFTR mutation, CFTR was corrected by either 3 µM VX809 for 24 h, 5 µM VX445 plus 18 µM VX661 for 48 h, or 5 µM VX445 plus 18 µM VX661 plus 1 µM VX770 for 48 h (all VX compounds were from Selleckchem). For these treatments, medium with drugs was replenished every 24 h.

In some experiments, non‐CF HBEs (nHBEs) were cultured using the Vertex method (Neuberger et al., 2011). Briefly, nHBE cells were isolated from human donor lung explants under a protocol approved by the Institutional Review Board. These cells were plated on Transwells and grown at an ALI in HBE differentiation medium containing 2% Ultroser G for 5–7 weeks.

### 2.2 Purification of bacterial SMase

Recombinant wild‐type (WT) and enzyme‐dead H322A *S. aureus* SMase were purified from BL21 (DE3) *E. coli* bacteria similarly to previously described (Cottrill et al., 2021; Stauffer et al., 2017). Briefly, bacteria were grown using kanamycin sulfate (Thermo Fisher) selection and IPTG induction. Bacteria were isolated by centrifugation, then lysed by sonication. The soluble material was isolated by centrifugation, then incubated overnight at 4°C with Roche cOmplete™ resin which was then washed and equilibrated according to the manufacturer's protocol. The resin was washed again, then incubated with elution buffer. Elution fractions were pooled, diluted to 4 mg/ml, and dialyzed in wash buffer using Spectrum Labs Float‐A‐Lyzers^®^ with 0.1 kDa molecular weight cutoff until the calculated concentration of imidazole was below 1 nM. Final protein concentration was determined by a Thermo Scientific Pierce™ BCA assay kit in accordance with the manufacturer's protocol. The H322A SMase mutant has been shown previously to have no detectible activity, and thus is an ideal control treatment (Cottrill et al., 2021; Stauffer et al., 2017). Note that in this study, SMase is added basolaterally, as we and others showed previously that WT SMase has no effect apically (Ito et al., 2004; Stauffer et al., 2017). We have shown that apical WT SMase does not hydrolyze apical sphingomyelin (Stauffer et al., 2017).

### 2.3 Lipidomics mass spectrometry

Lipidomics mass spectrometry was performed as described previously (Cottrill et al., 2021). All chemical reagents in this section are purchased from Thermo Fisher, unless otherwise noted. Briefly, cells were collected from Transwell filters by scraping, pelleted by centrifugation, flash frozen with liquid nitrogen, and stored at −80°C. Pellets were resuspended in 100 μl of isopropyl alcohol and subjected to three freeze–thaw cycles, then dried. Standards were obtained from Avanti Polar Lipids, Inc. The stock solution included the following standards, listed with their final target concentration diluted in Optima™ chloroform: 160 µg/ml 15:0–18:1(d7); 5 µg/ml 15:0–18:1(d7) phosphatidylethanolamine; 5 µg/ml 15:0–18:1(d7) phosphatidylserine; 30 µg/ml 15:0–18:1(d7) phosphatidylglycerol; 10 µg/ml 15:0–18:1(d7) phosphatidylinositol; 25 µg/ml 18:1(d7) lysophosphatidylcholine; 5 µg/ml 18:1(d7) lysophosphatidylethanolamine; 350 µg/ml 18:1(d7) cholesterol ester; 10 µg/ml 15:0–18:1(d7) diacylglycerol; 55 µg/ml 15:0–18:1(d7)‐15:0 triglyceride; 30 µg/ml 18:1(d9) sphingomyelin; and 100 µg/ml cholesterol (d7). The reconstitution solution was prepared by diluting the stock solution and deuterated ceramide LIPIDOMIX™ mass spec standard (d18:1–d7/16:0, d18:1–d7/18:0, d18:1–d7/24:0, d18:1–d7/24:1(15Z)) to 1.66% and 0.22% v/v, respectively, in isopropanol. Dried samples were reconstituted in this solvent, which has been described previously (Cottrill et al., 2021). Reconstituted samples were sonicated and centrifuged, with the supernatant stored at 4°C until analysis. For quality control purposes, a pooled sample and a sample blank were created.

Ultra‐performance liquid chromatography coupled with mass spectrometry was performed using a Vanquish system, an Accucore C30 column (2.1 × 150 mm, 2.6 µm particle size), and an Orbitrap ID‐X Tribrid mass spectrometer system. The method has been described previously (Cottrill et al., 2021). Experiments were performed by acquiring mass spectra in a data‐dependent acquisition fashion. Tandem mass spectrometry data were collected with a resolution of 120,000 and the dd‐MS2 data were collected at a resolution of 30,000, an isolation window of 0.8 *m*/*z*, and a cycle time of 2 s. Stepped normalized collision energies of 15, 30, and 45 were used to fragment selected precursors in the HCD cell prior to analysis in the Orbitrap. Dynamic exclusion was set at 2.5 s. Ions with charges >2 were omitted.

Data acquisition and processing were carried out using Compound Discoverer™ V3.0. After processing, peak areas were scaled by the median peak area of the sample. Annotation of the dataset was achieved by MS2 spectral matching to a local spectral database, built from curated experimental data. In addition, accurate mass, retention time, and isotopic pattern were matched to database entries.

Peak areas were normalized to a blank tube and the median peak intensity of all identified features. Data are reported either in bar graphs with the normalized peak area on the *y*‐axis, or as volcano plots with the −log (unadjusted *p* value) on the *y*‐axis and the difference in the normalized peak intensities on the *x*‐axis. A 5% false discovery rate was applied using the two‐stage step‐up method of Benjamini, Krieger, and Yekutieli (Benjamini et al., 2006), as recommended by Prism (GraphPad Software, Inc.). Statistical values were considered significant if both the *p* value and the *Q* value were less than 0.05. In the volcano plots, points that had a significant *p* value but not a significant *Q* value are identified by open circles, rather than filled circles.

### 2.4 Short‐circuit current measurements

Short‐circuit current experiments using an Ussing chamber were performed as described previously (Cui et al., 2019; Cottrill et al., 2021; Stauffer et al., 2017) using equipment from Physiologic Instruments. Acquire & Analyze software was used to collect data. The amplifier was set to voltage clamp at 0 mV to record short‐circuit currents. Bath buffer was prepared according to the protocol described previously (Ito et al., 2004; Cottrill et al., 2021). Normal chloride buffer (basolateral) was (in mM) 115 NaCl, 5 KCl, 1 MgCl_2_, 2 CaCl_2_, 10 glucose, 10 HEPES, 25 NaHCO_3_, pH 7.4. Low chloride buffer (apical) was (in mM) 115 Na gluconate, 5 KCl, 1 MgCl_2_, 4 CaCl_2_, 10 glucose, 10 HEPES, 25 NaHCO_3_, pH 7.4. Chambers were bubbled with 95:5% O_2_:CO_2_ and maintained at 37°C during experiments.

Cells were stabilized for 30 min prior to treatment with 1 µg/ml WT or enzyme‐dead H322A (mutant) SMase basolaterally for 30 min. To inhibit ENaC‐mediated sodium currents, 20 µM amiloride was added apically. To generate cAMP to stimulate CFTR, 0.01–10 µM forskolin was added. To potentiate CFTR currents, 1 µM VX770 was added. To inhibit CFTR, 10 µM INH172 was added apically. To inhibit ceramidase (CDase), 4 µM ceranib‐1 (Tocris) was added. To inhibit AMPK, 20 µM BML‐275 (Tocris) was added. The currents elicited by each treatment were calculated as the average of the final 10 s prior to the next treatment.

### 2.5 Analysis of surface expression of CFTR in nHBEs

Cells first were washed with Dulbecco's PBS with calcium and magnesium (DPBS), then equilibrated in normal chloride buffer, treated with 1 μg/ml SMase basolaterally for 30 min, then with 20 μM amiloride apically, and lastly treated with 10 μM forskolin. Cells were washed with ice‐cold DPBS and incubated with 0.5 mg/ml cell‐impermeable EZ‐Link™ Sulfo‐NHS‐SS‐Biotin (Thermo Fisher) apically on ice for 45 min. The biotinylation solution was removed, and excess Sulfo‐NHS‐SS‐Biotin was quenched with ice‐cold solutions of 50 mM Tris (pH 7.4), 100 mM glycine in 50 mM Tris, and 1 mM oxidized glutathione (Acros Organics) in DPBS. Cells were scraped from the filter and lysed for 30 min on ice in RIPA buffer (Boston BioProducts) with 3 mM EDTA and 1:100 protease inhibitor cocktail specific for CFTR (5 mg/ml AEBSF, 250 μg/ml chymostatin, 500 μg/ml E64, 1.25 mg/ml leupeptin, 1.25 mg/ml pepstatin (VWR), 17.4 mg/ml PMSF (Amresco), 250 μg/ml bestatin, and 36 mg/ml benzamidine) (Pollock et al., 2014). Cell debris was pelleted at 5000*g* for 5 min at 4°C, and the supernatant was retained as the total cell lysate. This cell lysate was incubated overnight at 4°C with Protein A/G beads (Santa Cruz) coupled to CFTR antibody 24‐1 (R&D) according to the manufacturer's protocol. Beads were pelleted at 1000*g* for 1 min, washed with DPBS three times, and finally protein was eluted in 2× sample buffer without any reducing agent at 37°C for 30 min.

### 2.6 Neutrophil transmigration

Neutrophils were isolated from blood of non‐CF donors under a protocol approved by the Emory University Institutional Review Board, and transmigrated through H441 epithelial cell monolayers using leukotriene B4 (LTB4) as a chemoattractant on the apical side, as described previously (Forrest et al., 2018).

Briefly, 250,000 H441 cells were cultured on Alvetex^®^ membranes (REPROCELL) coated with 3 mg/ml of rat tail collagen I, with DMEM + 10% FBS + 1% penicillin/streptomycin (pen/strep) + 1% l‐glutamine. The next day, cells were transitioned to ALI by removing apical medium and replacing basolateral medium with DMEM/F12 + 2% Ultroser G (Crescent Chemicals) + 1% pen/strep + 1% l‐glutamine. Neutrophil transmigration assays were performed 14 days after establishing H441 cells at ALI.

For neutrophil isolation, blood was collected into K2‐EDTA tubes, layered onto PolymorphPrep™ (Progen), and then centrifuged at 400*g* for 45 min at 21°C. The neutrophil band was collected with a Pasteur pipet, then washed in 0.45% NaCl, and centrifuged at 800*g* for 5 min at room temperature. Remaining erythrocytes were lysed by hypotonic shock. Neutrophils were resuspended in RPMI medium + 10% FBS, then counted and assessed for purity and viability with ethidium bromide + acridine orange staining. Our experiments showed >95% viability and <1% contamination of the neutrophil sample with other cells.

For transmigration, filters with H441 cells were inverted, LTB4 (100 nM) was added to the bottom RPMI medium (the apical side of the cells), and neutrophils (1.5 million/well) were added to the top RPMI medium (basolateral side of the cells). Transmigration was allowed to progress for 6 h at 37°C in a cell culture incubator with 5% CO_2_, at which point transmigrated neutrophils were collected from the apical medium by centrifugation at 800*g* for 10 min at 4°C. Cells were then counted and assessed for viability and epithelial cell contamination. Our experiments showed >95% viability and <1% contamination of the neutrophil sample with epithelial cells.

Where indicated, transmigrated neutrophils were allowed to condition medium (RPMI + 10% FBS, 4 million cells/ml) for 4 h at 37°C in a cell culture incubator. Conditioned medium was collected and centrifuged at 800*g* for 5 min at 4°C to separate out the neutrophils. The cell‐free supernatant (conditioned medium) had protease inhibitor cocktail and EDTA added, then was flash frozen in liquid nitrogen, and stored at −80°C for further analysis. The neutrophil pellet was resuspended, and neutrophils were counted and evaluated for cell death. Our experiments showed ~7% cell death after 4 h of incubation in conditioning medium. Neutrophils then were re‐pelleted and flash frozen in liquid nitrogen, then stored at −80°C for further analysis. For analysis, they were lysed in RIPA buffer with protease inhibitor cocktail and EDTA.

### 2.7 Western blot analysis

For analysis of acid‐SMase in HBEs experiments, sample buffer and 48 mM DTT were added to the samples. For NHS‐SS‐biotin experiments and analysis of acid‐SMase in neutrophils, no DTT was added, preventing reduction of disulfide bonds. Samples then were boiled at 95°C for 10 min. Samples then were run on a Bio‐Rad Mini‐PROTEAN TGX (4%–15%) gel. The gel was transferred onto a nitrocellulose membrane (Bio‐Rad, 0.2 μM), which then was blocked in Licor^®^ Intercept™ buffer. All subsequent probing agents were maintained in blocking buffer with 0.1% Tween 20. Blots with Licor secondary antibodies were imaged on a Licor^®^ Odyssey.

Total cellular CFTR in the cell lysate sample was evaluated by performing a Ponceau S stain prior to blocking the nitrocellulose membrane, followed by a PBS wash. The membrane then was washed, blocked, and incubated with 1:1500 anti‐CFTR 596 antibody (University of North Carolina) at 4°C overnight. The blot then was washed three times in PBS with 0.1% Tween 20 and incubated with 1:10,000 Licor^®^ goat anti‐mouse 680 antibody for 1 h at room temperature. The blot was washed and again imaged to evaluate total CFTR in the eluate. Total CFTR was normalized to two prominent Ponceau‐stained bands.

Biotinylated surface CFTR was evaluated using a Licor^®^ IRDye^®^ 680LT Streptavidin probe incubated at 1:5000 for 1 h at room temperature. The blot was imaged then stripped using Licor^®^ NewBlot™ Nitro Stripping buffer according to the manufacturer's protocol. The blot was reimaged to confirm sufficient removal of the original probe, re‐blocked, and the protocol for CFTR staining was followed as described in the previous paragraph.

Acid‐SMase also was analyzed in nHBEs and cfHBEs. Basolateral medium was collected 72 h after addition of fresh medium to the cells, except in the case of cfHBEs treated with correctors, in which the medium was changed every 24 h. Cells were collected and pooled from six Transwells and lysed as indicated above. Acid‐SMase and total protein were evaluated in both lysates and medium. Cellular and secreted acid‐SMase were evaluated by a similar protocol as above using 1:200 anti‐SMPD1 AF5348 antibody (R&D) overnight at 4°C, followed by 1:5000 Licor^®^ donkey anti‐goat 680 antibody for 1 h at room temperature. Total protein was probed with Ponceau S staining. In the cell lysate lanes, acid‐SMase density was normalized to an arbitrarily chosen prominent Ponceau‐stained band. In the basolateral medium lanes, acid‐SMase density was normalized to the most prominent Ponceau‐stained band, likely corresponding to BSA at approximately 66 kDa.

ImageJ software was used to conduct densitometry analysis on the appropriate bands of interest. Biotinylation bands of interest were determined by visual alignment with the corresponding CFTR bands.

### 2.8 Statistical analyses

Data were exported to and processed in Excel, unless otherwise noted. Statistical analyses were conducted and graphs were made using GraphPad Prism software, with α set at 0.05 according to common practice. Data were excluded based on Grubb's outlier tests with α set at 0.05 or in cases of technical experimental issues. In all cases, data are represented as the mean and standard deviation. In many cases, the individual data points are also plotted. Specific statistical tests as well as the significance values are listed in the figure legends, but included unpaired, two‐tailed *t*‐tests; multiple *t*‐tests without correction; and two‐way ANOVAs, utilizing the Tukey correction recommended by Prism if multiple comparisons were used.

## 3 RESULTS

### 3.1 Inhibiting CDases with ceranib‐1 affects CFTR currents similar to WT SMase

We have shown previously that acute basolateral WT SMase treatment decreases transepithelial CFTR currents in non‐CF bronchial epithelial cells (nHBEs), tracheal epithelial cells (nHTEs), and mixed populations of nHBEs and nHTEs (nHAEs) (Cottrill et al., 2021; Stauffer et al., 2017). We sought to determine whether the decreased CFTR currents after WT SMase treatment were due to decreased sphingomyelin, increased ceramide, or increased ceramide derivatives such as sphingosine or sphingosine‐1‐phosphate. To confirm that any ceramide breakdown product was not the cause for reduced CFTR currents following WT SMase treatment, we minimized the conversion of ceramide into sphingosine by treating nHAEs acutely with the CDase inhibitor ceranib‐1 while evaluating short‐circuit currents.

**FIGURE 2:**
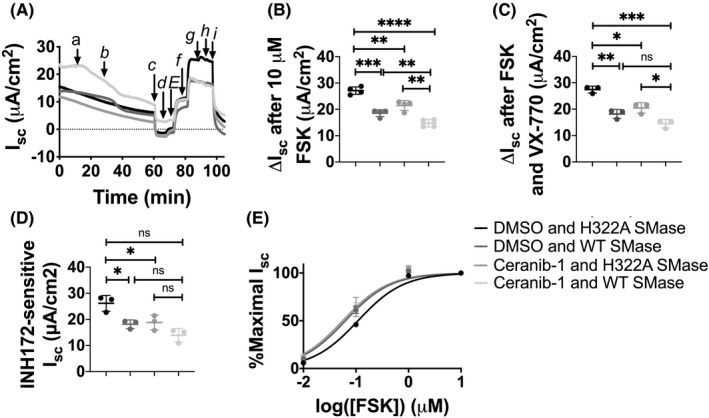
Inhibiting CDases with ceranib‐1 caused a decrease in CFTR currents and enhanced the WT SMase‐mediated inhibition of CFTR. (A) A representative example of an Ussing chamber trace of area‐corrected short‐circuit currents from nHAEs is shown. Cells stabilized for 5–10 min, at which point (a) 4 µM ceranib‐1 or an equivalent volume of DMSO was added. At the 30‐min timepoint, (b) 1 µg/ml enzyme‐dead H322A (with DMSO control, black, *n* = 3–4; with ceranib‐1 treatment, medium gray, *n* = 3–4) or WT SMase (with DMSO control, dark gray, *n* = 3–4; with ceranib‐1 treatment, light gray, *n* = 3–4) was added basolaterally. After 30 min, (c) 20 µM amiloride was added apically. Then, (d–g) 0.01, 0.1, 1, and 10 µM forskolin was added. Currents were potentiated by (h) 1 µM VX770, and finally were inhibited by (i) 10 µM INH172. Changes in current were evaluated by unpaired two‐tailed *t*‐tests. (B) The change from the post‐amiloride current to the current after the final dose of forskolin (forskolin‐elicited) was decreased by WT SMase alone (dark gray vs. black, ***p = 0.0010) and ceranib‐1 alone (medium gray vs. black, **p = 0.0029). Ceranib‐1 did not prevent WT SMase‐mediated decreases in currents (light gray vs. black, ****p < 0.0001), but rather enhanced them (light gray vs. dark gray, **p = 0.0076). In addition, WT SMase enhanced the ceranib‐1‐mediated decreases in current (light gray vs. medium gray, **p = 0.0013). (C) The change from the post‐amiloride current to the current after VX770 (VX770‐potentiated) was decreased by WT SMase alone (**p = 0.0018) and ceranib‐1 alone (**p = 0.0073). Ceranib‐1 did not prevent WT SMase‐mediated decreases in currents (***p = 0.0001). Significance was not quite reached to determine that ceranib‐1 enhanced WT SMase‐mediated decreases in current (*p* = 0.0529), but WT SMase did enhance ceranib‐1‐mediated decreases in current (*p = 0.0156). (D) The change from the post‐VX770 current to the current after INH172 (IN172‐sensitive) was reduced by WT SMase alone (*p = 0.0157) and ceranib‐1 alone decreased currents (*p = 0.0377). Lastly, (E) forskolin dose–response curves were generated by normalizing forskolin‐elicited currents to the current elicited by the maximum concentration of forskolin within each replicate, and then fitting points with a nonlinear fit of log (agonist) versus normalized response using Prism. A comparison of fits determined that one equation could fit the data for DMSO + H322A SMase, and a different equation fit the data for all the other treatments. This indicates that CFTR is in one activatable state under control conditions, but a different and potentially common activatable state under the other conditions.

First, we replicated our previous results showing that WT SMase decreases transepithelial CFTR currents (Figure 2A). Specifically, in nHAEs, 30‐min treatment with 1 µg/ml WT SMase decreased the maximum forskolin‐elicited currents from 27.1 ± 1.4 µA/cm^2^ to 18.6 ± 1.3 µA/cm^2^ (approximate 31.5% decrease), decreased VX770‐potentiated currents from 27.4 ± 1.3 µA/cm^2^ to 18.2 ± 1.7 µA/cm^2^ (approximate 33.6% decrease), and decreased INH172‐sensitive current from 26.2 ± 3.0 µA/cm^2^ to 18.1 ± 1.6 µA/cm^2^ (approximate 30.6% decrease), as compared to the enzyme‐dead H322A SMase control (Figure 2B–D). Preventing the conversion of ceramide into sphingosine (and consequently other derivatives) by treating with ceranib‐1 did not mitigate WT SMase‐mediated decreases in forskolin‐elicited, VX770‐potentiated, or INH172‐sensitive CFTR currents (Figure 2B–D). This suggests that a ceramide derivative is not the cause of SMase‐mediated inhibition of CFTR currents.

In fact, ceranib‐1 treatment exacerbated WT SMase‐mediated decrease of forskolin‐elicited currents, relative to WT SMase or ceranib‐1 treatment alone (Figure 2B). Note that while this trend remained in the VX770‐potentiated and INH172‐sensitive currents, the results did not reach statistical significance, likely due to a smaller number of replicates. Relatedly, though, 1 h ceranib‐1 treatment alone decreased the maximum forskolin‐elicited currents to 21.5 ± 1.9 µA/cm^2^ (approximate 20.8% decrease), decreased VX770‐potentiated currents to 20.5 ± 2.0 µA/cm^2^ (approximate 25.3% decrease), and decreased INH172‐sensitive current to 18.8 ± 2.8 µA/cm^2^ (approximate 28.0% decrease), as compared to the control H322A SMase control (Figure 2B–D). This was similar to the effects of control WT SMase treatments.

These data indicate that ceranib‐1 and WT SMase each decrease CFTR currents, but the question remained whether they utilize the same mechanism or different mechanisms to facilitate this reduction of current. Analysis of the forskolin‐mediated activation of CFTR indicated that WT SMase, ceranib‐1, and the combination of these treatments shifted the dose–response curve left relative to the control treatment (Figure 2E). Interestingly, data from all three of these treatments could be fit with the same curve, suggesting that the ability to activate CFTR (dependent on adenylyl cyclase, PKA, and ATP) was the same following SMase treatment, ceranib‐1 treatment, and dual treatment of SMase and ceranib‐1. Given that dual SMase and ceranib‐1 treatment did not further shift the dose–response curve compared to either treatment alone, it is likely that WT SMase and ceranib‐1 decrease CFTR currents via the same mechanism. However, it is still possible that they elicit their effects via distinct mechanisms.

### 3.2 Ceranib‐1 affects total cellular dihydroceramides, as does SMase

**FIGURE 3:**
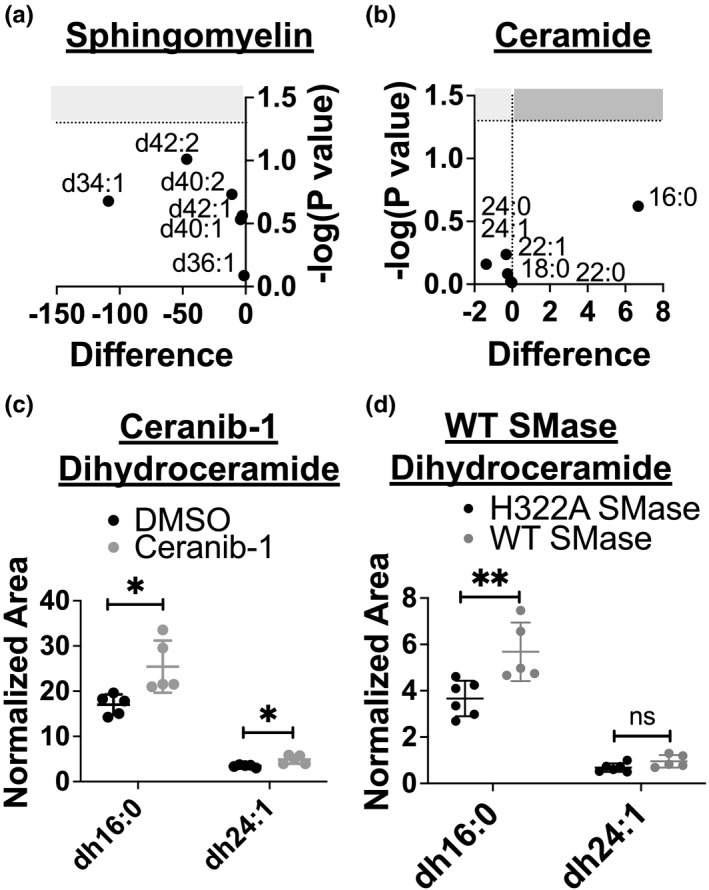
Lipidomics mass spectrometry analysis indicated that ceranib‐1 increased dihydroceramides. The (a) sphingomyelin and (b) ceramide data were analyzed by multiple *t*‐tests, which indicate that none of the total cellular levels of those lipids were affected by ceranib‐1 treatment. These data are plotted as volcano plots, with the difference in normalized peak area on the *x*‐axis, and the −log (*p* value) on the *y*‐axis. However, (c) two dihydroceramide species were increased by ceranib‐1 treatment (gray, *n* = 5) as compared to DMSO control (black, *n* = 5) (dh16:0, *p = 0.0161; dh24:1, *p = 0.0105). (d) WT SMase (gray, *n* = 5) increased the dh16:0 dihydroceramide compared to the enzyme‐dead H322A SMase control (black, *n* = 6) (**p = 0.0095), but not quite the dh24:1 species (*p* = 0.0780)

In order to determine if WT SMase and ceranib‐1 treatment generated similar changes in sphingolipids, we performed lipidomics mass spectrometry analysis. We have shown previously that WT SMase treatment decreases all sphingomyelins and increases all ceramides of interest (Cottrill et al., 2021). Analysis of nHAEs acutely treated with ceranib‐1 indicated that ceranib‐1 caused no significant effect on bulk cellular sphingomyelins or ceramides (Figure 3a,b). Localized effects on sphingomyelin and ceramide cannot be ruled out, though, and future experiments should evaluate plasma membrane sphingolipids specifically. However, it was determined that ceranib‐1 significantly increased two dihydroceramides (dh16:0 and dh24:1) which correspond to the two highest abundance ceramides (Figure 3c). Interestingly, WT SMase treatment also increased the dh16:0 dihydroceramide in nHBEs, and almost significantly increased the dh24:1 dihydroceramide (Figure 3d). Thus, ceranib‐1 shares a mechanism with WT SMase either by increasing dihydroceramides, or by increasing ceramides in a localized manner undetectable by whole‐cell lipidomics analysis, or both.

### 3.3 No decrease in CFTR surface expression was detected following SMase treatment

To continue to explore how WT SMase decreases transepithelial CFTR currents, we assessed potential effects on surface expression. Utilizing the cell‐impermeable Sulfo‐NHS‐SS‐Biotin compound, we labeled all apical membrane proteins at the surface of nHBEs to compare the abundance of surface CFTR following treatment with either H322A or WT SMase.

**FIGURE 4:**
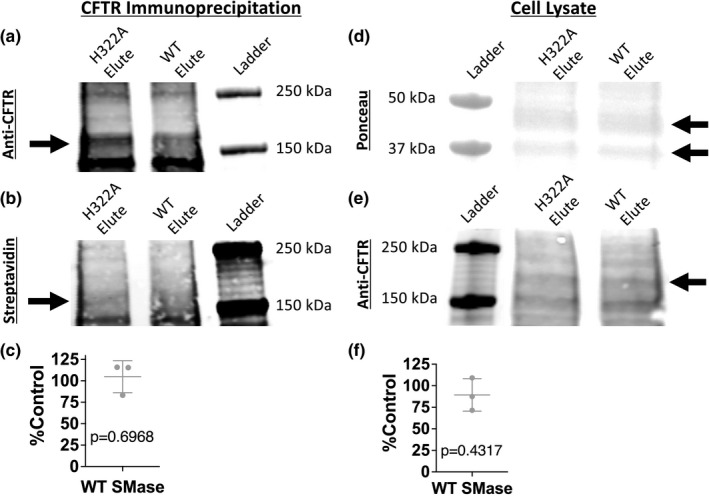
Surface biotinylation followed by a CFTR immunoprecipitation indicated that SMase did not affect the abundance of total cellular or surface expressed CFTR. Representative blots for (a) CFTR and (b) biotinylation in samples following CFTR immunoprecipitation are shown. The protein band for CFTR is indicated by an arrow. (c) Densitometry analysis was performed, and the relative amount of biotinylated surface CFTR between WT and H322A SMase‐treated samples was normalized to the relative amount of CFTR (*n* = 3). A one‐sample *t*‐test indicates no significant difference from 100% (*p* = 0.6968), suggesting that WT SMase does not affect the surface CFTR abundance. Representative blots for (d) total protein (ponceau) and (e) CFTR in the cellular lysate are shown. The two protein bands used for normalization are indicated by arrows, as is the band for CFTR. (f) Densitometry analysis was performed, and the relative amount of cellular CFTR between WT and H322A SMase‐treated samples was normalized to the relative density of the ponceau bands, averaged (*n* = 3). A one‐sample *t*‐test indicates no significant difference from 100% (*p* = 0.4317), suggesting that WT SMase does not affect the cellular CFTR abundance

We used CFTR immunoprecipitation to evaluate the levels of surface biotinylated CFTR, as isolation of all biotinylated proteins using streptavidin beads did not result in an identifiable CFTR band. In these immunoprecipitation samples, analyzed in the absence of DTT to prevent reduction of the disulfide bond in NHS‐SS‐biotin, biotinylated CFTR density was normalized to total CFTR density (Figure 4a,b). WT SMase caused surface CFTR to be 104% ± 19% of the H322A SMase control, which was not statistically different from 100% (Figure 4c). Note that these immunoprecipitation experiments were also performed with NHS‐LC‐Biotin and showed similar results for surface CFTR (*data not shown*, *n* = 4, 122 ± 44%, *p* = 0.3970). To confirm that total CFTR abundance was not affected by SMase treatment, given that this was the normalization control, we also evaluated total cellular CFTR in the cell lysate samples. The CFTR band was normalized to the two prominent ponceau stain bands (Figure 4d,e). These normalized data indicated that total cellular CFTR was unaffected by WT SMase treatment (30–45 min, 1 μg/ml, 37°C), with WT SMase being 89.37 ± 19% of the H322A SMase control (Figure 4f), suggesting that normalization to total CFTR in the immunoprecipitation experiments did not bias the results.

Overall, these data indicate that WT SMase did not cause a decrease in CFTR abundance at the apical surface of nHBEs. This is consistent with our previous experiments in *Xenopus* oocytes, which indicated no effects of SMase on surface expression of CFTR. This is even further supported by the shift in the forskolin dose–response curve following SMase treatment, as decreased surface expression of CFTR would not cause a shift in the dose–response curve since the currents are normalized to the maximum current and a change in surface expression does not affect the activatability of CFTR. Altogether, these data rule out decreased CFTR surface expression as the means by which basolateral WT SMase decreases apical membrane conductance in nHBEs (Stauffer et al., 2017).

### 3.4 AMPK is not involved in the WT SMase‐mediated decrease in transepithelial CFTR currents

**FIGURE 5:**
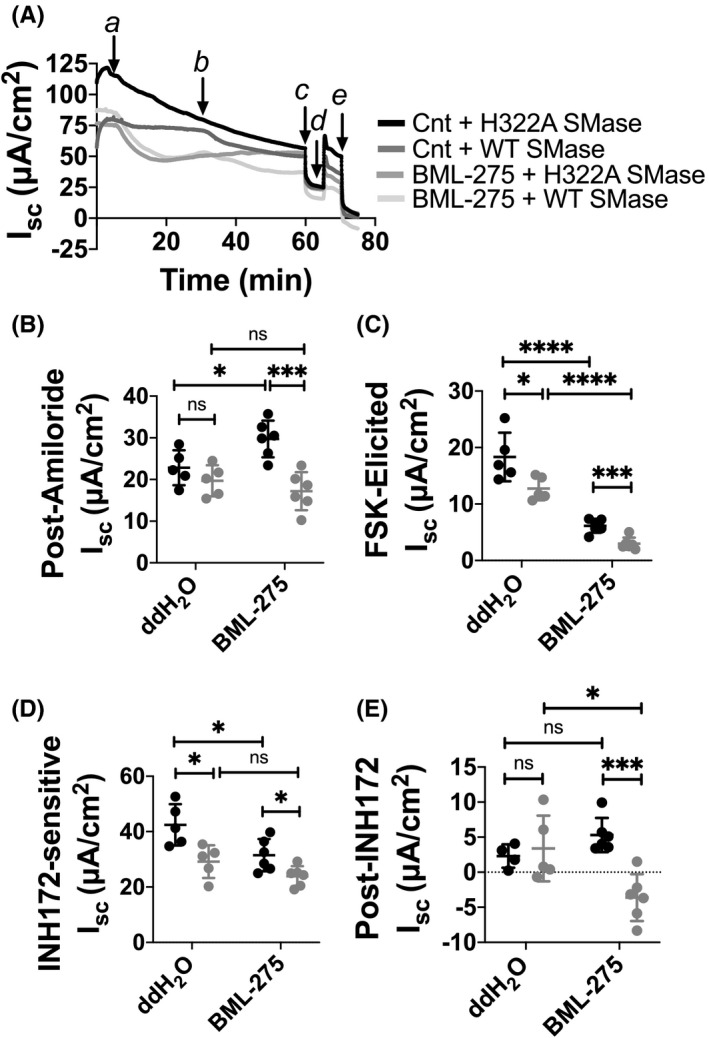
Inhibiting AMPK does not prevent WT SMase‐mediated decreases in transepithelial CFTR currents but does have other effects on currents. (A) An example area‐corrected short‐circuit current trace is shown of nHBEs treated with either (a) 20 μM BML‐275 or vehicle control (Cnt or ddH2O, double‐distilled water), then (b) 1 μg/ml either H322A or WT SMase basolaterally for 30 min. (c) ENaC was inhibited with 20 μM amiloride apically, then (d) CFTR was activated with 10 μM forskolin. Lastly, (e) CFTR was inhibited with 10 μM INH172 apically. All data were analyzed by unpaired, two‐tailed *t*‐tests. For these short‐circuit current analyses, *n* = 4–6 for each group. (B) For the post‐amiloride current, in the BML‐275 group WT SMase caused a significant decrease (***p = 0.0007), a phenomenon not seen in the control treatment (*p* = 0.2454). However, BML‐275 itself increased the post‐amiloride current in the H322A SMase group (*p = 0.0267). (C) For the forskolin‐elicited current, the control and BML‐275 groups both showed a WT SMase‐mediated decrease in current (*p = 0.0302; ***p = 0.0008). However, BML‐275 itself decreased the forskolin‐elicited currents in both the H322A and WT SMase groups (****p < 0.0001). (D) For the INH172‐sensitive current, both the control and BML‐275 groups showed a WT SMase‐mediated decrease in current (*p = 0.0144; *p = 0.0218). However, BML‐275 itself decreased the INH172‐sensitive currents in the H322A SMase group (*p = 0.0229). (E) For the post‐INH172 current, within the BML‐275 group, there was a significant decrease caused by WT SMase (***p = 0.0007), a phenomenon not seen in the control group (*p* = 0.2454). However, BML‐275 itself caused an almost significant increase in the post‐INH172 current in the H322A SMase group (*p* = 0.0667). Conversely, BML‐275 caused a significant decrease in the post‐INH172 currents in the WT SMase group (*p = 0.0177)

In *Xenopus* oocytes, we found that intracellular components were necessary for a decrease in CFTR‐mediated current following WT SMase treatment, indicating that a signaling mechanism was involved (Stauffer et al., 2017). Based on the above surface expression data in nHBEs indicating a lack of SMase‐induced internalization of CFTR, it is likely that a signaling mechanism is involved in nHBEs as well. Both ceramide and dihydroceramide are known to activate AMPK (Ji et al., 2010; Siddique et al., 2013), which phosphorylates the CFTR R‐domain at the inhibitory S737 and S768 sites (Hallows et al., 2000; Kongsuphol et al., 2009). Thus, we hypothesized that AMPK activation via the generation of ceramide and/or dihydroceramide was involved in WT SMase‐mediated decreases in transepithelial CFTR current. In order to test this, we utilized the AMPK inhibitor dorsomorphin dihydrochloride (BML‐275) (Figure 5).

We found that inhibiting AMPK with BML‐275 did not prevent WT SMase‐mediated reductions in the forskolin‐elicited or INH172‐sensitive currents in nHBEs (Figure 5). Specifically, for the forskolin‐elicited currents, within the control group WT SMase caused a 31% reduction of currents from 18.7 ± 4.9 µA/cm^2^ to 12.9 ± 1.9 µA/cm^2^, whereas in the BML‐275 group WT SMase caused a 51% reduction of currents from 6.1 ± 1.2 µA/cm^2^ to 3.0 ± 1.1 µA/cm^2^. For the INH172‐sensitive currents, within the control group WT SMase caused a 26% reduction of currents from 42.7 ± 8.6 µA/cm^2^ to 31.5 ± 8.0 µA/cm^2^, whereas in the BML‐275 group WT SMase caused a similar 22% reduction of currents from 30.6 ± 4.6 to 23.8 ± 3.6 µA/cm^2^. These data strongly suggest that AMPK is not involved in the signaling pathway by which WT SMase decreases transepithelial CFTR currents. However, it is important to note that BML‐275 had other effects on the cells, such as increasing the post‐amiloride baseline current (Figure 5B), decreasing the forskolin‐elicited current (Figure 5C), decreasing the INH172‐sensitive current (Figure 5D), and variably affecting the post‐INH172 current (Figure 5E); these findings suggest that the inhibitor was used at an effective concentration. While these effects must be considered when interpreting the data, we remain confident that inhibiting AMPK does not prevent WT SMase‐mediated decreases in CFTR currents in nHBEs.

### 3.5 Ceramide and dihydroceramide levels in cfHBEs compared to nHBEs

While some work already has been performed to identify ceramide and dihydroceramide imbalances in CF bronchial epithelial cells compared to non‐CF bronchial epithelial cells (Gardner et al., 2020; Hamai et al., 2009; Loberto et al., 2020), much of the data are conflicting. Thus, we thought it important to assess these imbalances within our own cells and to determine the potential impact of culture conditions that may explain differences in results between laboratories.

**FIGURE 6:**
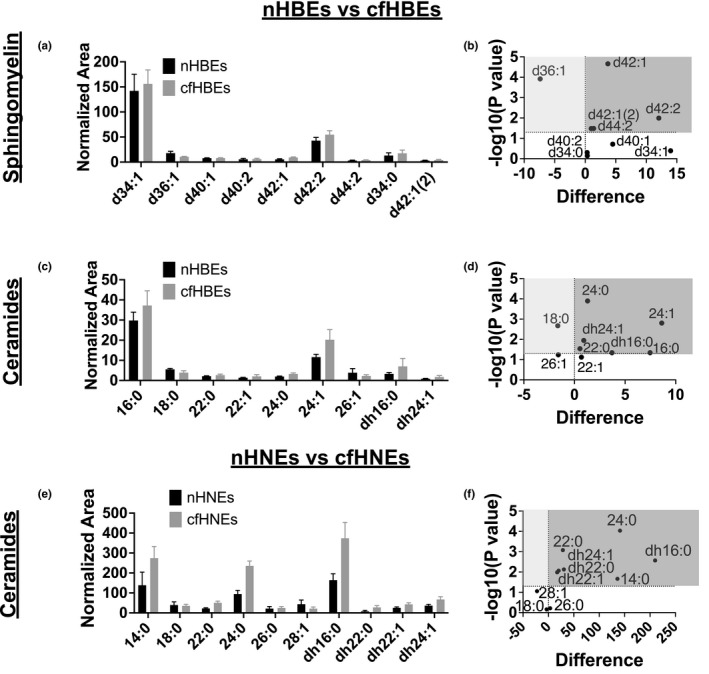
Bar graphs and volcano plots of sphingolipid species in HBEs and HNEs are shown. Sphingomyelin d42:1(2) refers to the second species of d42:1 sphingomyelin. For the volcano plots, anything in the dark gray area was increased in the CF group, whereas anything in the light gray area was decreased. Lipidomics analysis of a single biological replicate of cfHBEs (*n* = 8 Transwells) as compared to a single biological replicate of nHBEs (*n* = 6 Transwells) was used to evaluate (a and b) sphingomyelins as well as (c and d) ceramides and dihydroceramides. Many sphingomyelins were increased, though one was decreased in cfHBEs relative to nHBEs. Most ceramides were increased, one ceramide was decreased, and two dihydroceramides were increased. (e and f) Lipidomics analysis of a single biological replicate of cfHNEs (*n* = 4 Transwells) as compared to a single biological replicate of nHNEs (*n* = 4 Transwells) indicated that most ceramides were increased, and all dihydroceramides were increased in cfHNEs relative to nHNEs, similar to the phenomenon seen in HBEs

First, we analyzed nHBEs grown on T25 flasks for 7 days, Transwells for 8 days, and Transwells for 14 days to determine if ceramide or dihydroceramide levels changed over the course of differentiation. These data indicate that differentiation affects sphingolipid profiles, and that time on the Transwell is an important factor to control for when comparing cfHBEs to nHBEs (Figure S1). Keeping this in mind, we compared the sphingomyelin, ceramide, and dihydroceramide levels of multiple Transwells of a single biological replicate of cfHBEs (homozygous for ΔF508‐CFTR) to multiple Transwells of a single biological replicate of nHBEs (Figure 6a–d). In cfHBEs relative to nHBEs, four sphingomyelins were increased (d42:1, d42:1 (second species), d42:2, and d44:2). Based on the ceramides and dihydroceramides present, these likely correspond to 24:0 ceramide, dh24:1 dihydroceramide, 24:1 ceramide, and 26:1 ceramide, though this cannot be confirmed given our current methods. Simultaneous with these increases, a single sphingomyelin was decreased (d36:1). In cfHBEs relative to nHBEs, four ceramides were increased (16:0, 22:0, 24:0, and 24:1). These included the two most abundant ceramides, 16:0 and 24:1, and both long‐chain and very‐long‐chain ceramides. Both dihydroceramides also were increased (dh16:0, dh24:1), each corresponding to the most abundant ceramide species. Some of these increased ceramides and dihydroceramides likely correspond to the increased sphingomyelins. Specifically, 24:0 ceramide and dh24:1 dihydroceramides could each be one of the d42:1 sphingomyelins, and 24:1 ceramide could be d42:2 sphingomyelin. Simultaneous with these increases in ceramide, one ceramide was decreased (18:0), which likely corresponds to the one decreased sphingomyelin (d36:1). Importantly, broadly comparing the downregulated ceramide to the upregulated ceramides does not indicate a differential effect on long‐chain versus very‐long‐chain ceramides, as had been seen previously by another group (Veltman et al., 2021). Furthermore, the fact that both sphingomyelins and ceramides are largely increased in cfHBEs relative to nHBEs suggests that the increase in ceramides is not solely due to increased breakdown of sphingomyelin in cfHBEs.

When working with primary HBEs, it is important to keep in mind that they generally are obtained from lungs of patients with disease state advanced enough to warrant a lung transplant. We were interested in analyzing an airway cell type that could be collected at any stage of CF, and that could be age‐ and gender‐matched to truly “healthy” controls, rather than other non‐CF lung transplant cells. Thus, we evaluated sphingolipid imbalances in nHNEs from a 25‐year‐old female donor and cfHNEs from a 23‐year‐old female donor homozygous for the ΔF508‐CFTR mutation (Figure 6e–f). The most abundant sphingomyelins were a dihydrosphingomyelin (fully saturated) and an odd‐chained sphingomyelin (*data not shown*). As such, we did not think it appropriate to compare the sphingomyelins of HNEs to those of HBEs. Interestingly, the most prominent ceramides and dihydroceramides were slightly different in these HNEs than in the HBEs, though certainly less so than the sphingomyelins. Most notably, 14:0 and 24:0 were the most prominent ceramides rather than 16:0 and 24:1. Similarly to HBEs, though, lipidomics analysis revealed that cfHNEs, as compared to nHNEs, had increased levels of many ceramides (14:0, 22:0, and 24:0). This includes the two most prominent ceramides as well as long‐chain and very‐long‐chain ceramides. Note, also, that the 22:0 and 24:0 ceramides were increased in the cfHBEs as well. Along with these increases in ceramides, all four dihydroceramides (dh16:0, dh22:0, dh22:1, and dh24:1) were increased in cfHNEs. No ceramides were decreased. Again, this indicates no differential effect on long‐chain versus very‐long‐chain ceramides, as had been seen previously by another group (Veltman et al., 2021). Thus, when considering the ceramide and dihydroceramide data gathered in HBEs as well as HNEs, it appears that the general trend is an increase in ceramides and dihydroceramides in CF cells.

### 3.6 Effects of modulator therapy on sphingolipids in cfHBEs

Given our own data and previous literature (Gardner et al., 2020; Hamai et al., 2009; Loberto et al., 2020), cfHBEs clearly have increased ceramides and dihydroceramides compared to nHBEs. However, many CF patients are approved for treatment with CFTR modulators, so it is important to determine if these modulators can rescue sphingolipid imbalances.

**FIGURE 7:**
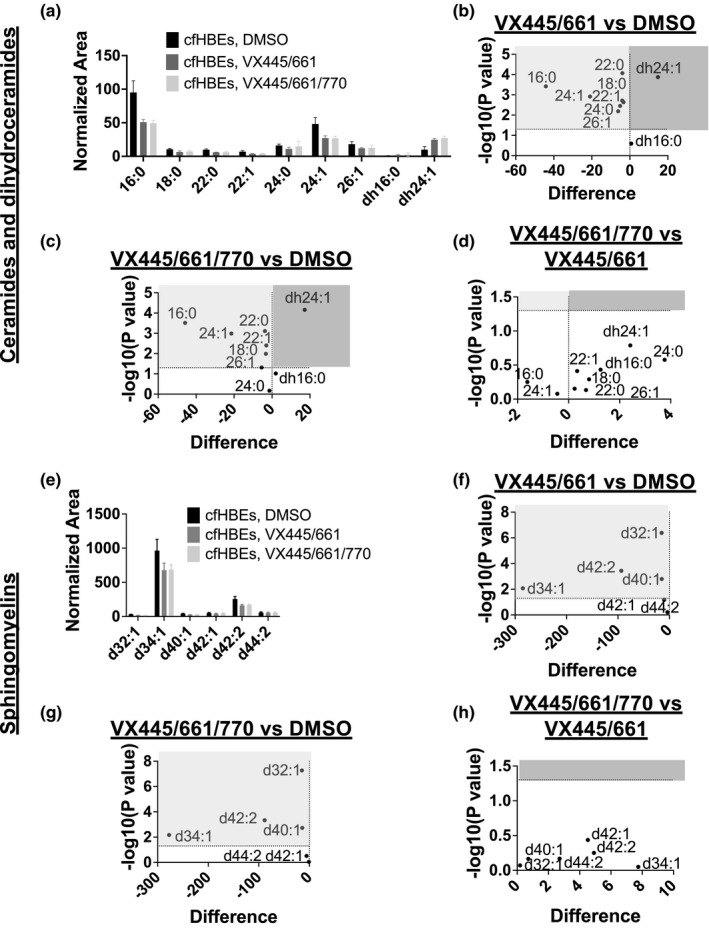
Lipidomics analysis indicates that the newest FDA‐approved correctors can affect the sphingolipid imbalances in cfHBEs. Any sphingolipid in a light gray box was significantly decreased by treatments, whereas any sphingolipid in a dark gray box was significantly increased by treatments. (a–d) In cfHBEs, 48 h treatment with VX445 + VX661 (*n* = 5) or VX445 + VX661 + VX770 (*n* = 5) decreased all ceramides of interest except for one ceramide in the VX445 + VX661 + VX770 treatment group, though no difference was found between treatments. Four of these ceramides were significantly increased in cfHBEs compared to nHBEs, so modulators corrected these imbalances. Simultaneously, VX445 + VX661 and VX445 + VX661 + VX770 treatments both increased one dihydroceramide, again with no difference between the two treatments. This dihydroceramide was already increased in cfHBEs as compared to nHBEs, indicating that treatment pushed this dihydroceramide even further away from the nHBE levels. (e–h) In regard to sphingomyelin, both VX445 + VX661 and VX445 + VX661 + VX770 decreased four sphingomyelins of interest, with no difference between the two treatments

We first treated cfHBEs with VX809 for 24 h and found no effects on ceramides or dihydroceramides (*data not shown*). We then treated cfHBEs either with VX445 + VX661 or with VX445 + VX661 + VX770 for 48 h (Figure 7). These treatments rescue either CFTR trafficking or CFTR trafficking and activity, respectively, allowing us to begin to distinguish the effects of accumulated unfolded protein as compared to a loss of CFTR activity. Lipidomics analysis of these cfHBEs indicated that, compared to DMSO‐treated cfHBEs, VX445 + VX661 decreased all ceramides of interest and increased one dihydroceramide (dh24:1) (Figure 7b). Treating cells with VX445 + VX661 + VX770 decreased all but one ceramide of interest (24:0) and increased the same dihydroceramide (Figure 7c). No significant difference was found between the two different treatments (Figure 7d).

In an effort to understand if this effect was due to decreased sphingomyelin breakdown, we evaluated the effects of these drugs on sphingomyelin levels as well (Figure 7e–h). Interestingly, VX445 + VX661 alone as well as VX445 + VX661 + VX770 for 48 h decreased four sphingomyelins (d32:1, d34:1, d40:1, and d42:2), with no difference between the two treatments. One of these sphingomyelins (d42:2) was identified above as being increased in cfHBEs relative to nHBEs. Given that these drugs decreased sphingomyelins as well as ceramides, it is unlikely that the decrease in ceramide is due to a decrease in conversion of sphingomyelin into ceramide. An alternative pathway that can explain a reduced presence of both sphingolipids must be involved and should be identified.

### 3.7 Imbalance of acid‐SMase in cfHBEs

Though the above lipidomics data suggest that increased breakdown of sphingomyelin alone cannot explain increased ceramide levels in CF airway cells, differential activity of acid‐SMase could at least be partially involved still. Previous literature has indicated elevated levels of SMase activity on the surface of cfHBEs compared to nHBEs, though the same study found no increase in cellular mRNA or protein levels of acid‐SMase (Gardner et al., 2020). Thus, we sought to evaluate cellular and extracellular levels of acid‐SMase protein in nHBEs and cfHBEs using western blot and densitometry analysis on a single biological replicate. Importantly, note that lysosomal and secreted acid‐SMase, though arising from the same gene, are distinct (Jenkins et al., 2011; Kornhuber et al., 2015; Qiu et al., 2003; Schissel et al., 1996, 1998).

Following normalization to Ponceau staining, these analyses indicated that cfHBEs had approximately 22% more cellular acid‐SMase (~70 kDa band) than nHBEs (Figure S2a,b), and about 11% more extracellular acid‐SMase (~57 kDa band) (Figure S2c,d). Confidence in our conclusions is enhanced by the finding that both total cellular and extracellular acid‐SMase levels were increased in cfHBEs. While increased acid‐SMase levels in cfHBEs could partially explain increases in some of the ceramide levels in cfHBEs compared to nHBEs, further studies blocking acid‐SMase activity would need to be performed to confirm this.

### 3.8 LTB4‐transmigrated neutrophils secrete acid‐SMase

Everything evaluated thus far has been in CF and non‐CF primary airway epithelial cells in an isolated laboratory setting. However, the actual lung environment is much more complicated. Endogenously, HBEs scaffold on top of basal cells and fibroblasts, and immune cells such as macrophages, neutrophils, and T cells are recruited through the HBEs into the airspace. Previous work has shown that macrophages, among many other cell types, secrete acid‐SMase (Schissel et al., 1996). However, no one to our knowledge has evaluated acid‐SMase secretion from neutrophils. Neutrophils are particularly important to the pulmonary pathophysiology of CF and are present in much higher abundance in CF airways than other immune cells (Giacalone et al., 2020). Thus, we resolved to determine the presence of cellular and secreted acid‐SMase in transmigrated neutrophils.

**FIGURE 8:**
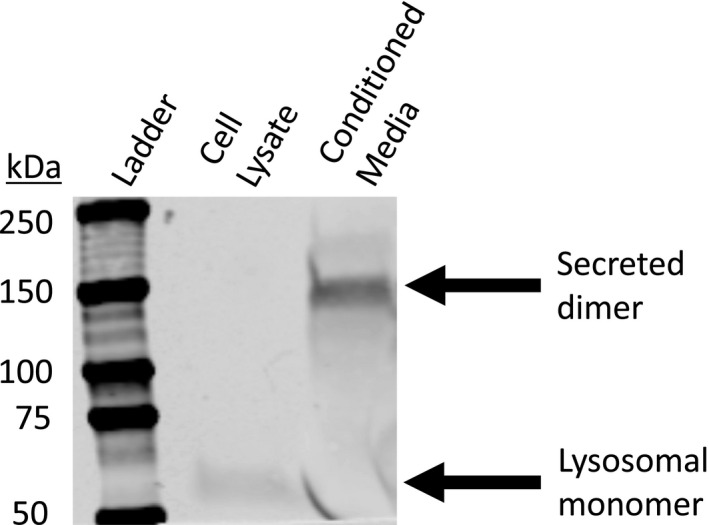
Neutrophils transmigrated through H441 cells by LTB4 stimulation, as described in Methods, were evaluated for cellular and secreted acid‐SMase after 4 h. In the left lane, a protein standard ladder is shown with labeled molecular weights. In the middle lane, cellular lysate of the transmigrated neutrophils shows lysosomal acid‐SMase around 60 kDa. In the right lane, conditioned medium shows secreted acid‐SMase dimer around 150 kDa. Transmigrated neutrophils clearly secrete acid‐SMase. DTT was not added in this experiment

Neutrophils were collected from subjects without CF and transmigrated across non‐CF epithelial cells in response to chemoattraction using LTB4, then allowed to condition the apical medium for 4 h. These cells and the medium they conditioned were then separated and analyzed for acid‐SMase (Figure 8). Acid‐SMase was clearly detectible in the cellular lysate of these neutrophils, at the approximate molecular weight expected for lysosomal acid‐SMase (~60 kDa). Furthermore, acid‐SMase was clearly detectible in the conditioned medium of these neutrophils, at the approximate molecular weight for dimerized secreted acid‐SMase (~150 kDa), since DTT was not added in this experiment. Thus, these results show that LTB4‐transmigrated neutrophils are an endogenous source of acid‐SMase in the lung environment. The effects of this secretion should be characterized in future work.

## 4 DISCUSSION

In this study, we found that inhibiting ceramide breakdown by treating nHAEs with ceranib‐1 decreased the forskolin dose–response curve similar to WT SMase, suggesting that these treatments decreased CFTR currents via the same mechanism. Long‐term treatment with ceranib‐1 increases ceramide levels and theoretically would not affect sphingomyelin or phosphocholine levels (Draper et al., 2011). Importantly, lipidomics analysis of nHBEs treated with ceranib‐1 for 1–2 h, as in our experiments, showed no apparent effect on total cellular sphingomyelins or ceramides.

Instead, we detected an increase in two dihydroceramides, one of which was also found to be increased by WT SMase treatment alone. Dihydroceramides are a step in the de novo ceramide synthesis pathway and are converted into ceramide in the ER by dihydroceramide desaturase (Siddique et al., 2015). Since both WT SMase and ceranib‐1 treatment increase these lipids, dihydroceramides could be the commonality between the inhibitory mechanisms of these two treatments. It is also possible that ceranib‐1 caused localized changes in ceramide that were not detected when evaluating total cellular lipids. Further experiments evaluating the specific changes in plasma membrane ceramides along with experiments acutely adding ceramide and dihydroceramide to the cells are necessary to determine if ceramide accumulation, dihydroceramide accumulation, both, or neither is the mechanism underlying WT SMase‐ and ceranib‐1‐mediated inhibition of CFTR.

The potential effects of ceramide and dihydroceramide on CFTR activity are relevant to many inflammatory lung diseases, and to CF in particular (Chakinala et al., 2019; Devlin et al., 2011; Ghidoni et al., 2015). Multiple groups have identified an increase in ceramides in CF airway cells as compared to non‐CF controls (Gardner et al., 2020; Loberto et al., 2020), though one group found an overall decrease in ceramides with a simultaneous increase in the ratio of long‐chain ceramides to very‐long‐chain ceramides (Veltman et al., 2021). Dihydroceramides also have been found to be increased in immortalized CF cell line models (Hamai et al., 2009). We report within this study that many ceramides and dihydroceramides are increased in cfHBEs compared to nHBE controls. We also found this phenomenon in cfHNEs as compared to age‐ and sex‐matched nHNE controls, which can be collected non‐invasively at any stage of CF and from healthy non‐CF donors. Evaluations on sphingolipids in cfHNEs have not been reported previously to our knowledge. Interestingly, in HBEs, this increase in ceramides was accompanied by an increase in sphingomyelins as well. This suggests that increased acid‐SMase activity alone cannot explain the increased ceramide levels, as decreased sphingomyelin levels would be expected in that case.

There are current FDA‐approved modulators to address the mistrafficking and reduced activity of CFTR, so it was important to evaluate the effects of these drugs on the sphingolipid profile of cfHBEs. In this study, we found that treatment with 24 h VX809 had no effect on sphingolipid profiles. This is consistent with previous work in CFBE41o‐cells (Loberto et al., 2020) but is not consistent with previous work in cfHBEs that shows a slight decrease in a ceramide species not found at a large abundance in the cfHBEs in this study (14:0) (Zheng et al., 2006).

We also found that 48 h VX445 + VX661 alone decreased all ceramides of interest. Importantly, effects of this treatment, which only facilitates rescue of misfolding and trafficking of ΔF508‐CFTR, were no different than effects of 48 h treatment with VX445 + VX661 + VX770, which facilitates rescue of misfolding, trafficking, and subsequent activity of ΔF508‐CFTR. The observed effects of VX445 + VX661 + VX770 on ceramides in cfHBEs are consistent with previous work in CFBE41o‐cells (Loberto et al., 2020) but is not consistent with previous work in cfHBEs which found that VX445 + VX661 + VX770 treatment increased total ceramide levels (Zheng et al., 2006). No previous work to our knowledge has separately evaluated the effects of these highly effective correctors alone on the sphingolipid profiles of homozygous ΔF508‐CFTR cfHBEs as compared to the correctors plus the potentiator VX770. Our results suggest that the unfolded protein response due to mistrafficked ΔF508‐CFTR may be the cause of ceramide imbalances, rather than a loss of CFTR function. Of note, though, is that modulator therapy further exacerbated the increase in one very‐long‐chain dihydroceramide (dh24:1) in cfHBEs, which could prove problematic if this dihydroceramide has detrimental effects on the cells. Furthermore, many sphingomyelins were decreased by VX445 + VX661 or VX445 + VX661 + VX770 treatment. This suggests that the decrease in ceramides caused by these treatments is not due to decreased acid‐SMase activity alone, as increased sphingomyelin levels would be expected in this case. Another mechanism must be involved.

In order to begin to understand if acid‐SMase could be involved in increased ceramides in cfHBEs, we evaluated acid‐SMase protein levels in cfHBEs and nHBEs and conditioned medium thereof, especially given that increased surface acid‐SMase activity has been identified previously in cfHBEs (Gardner et al., 2020). A single biological replicate of our cfHBEs showed increased protein levels of both cellular and secreted acid‐SMase as compared to nHBEs. While the increased total cellular protein levels of acid‐SMase in cfHBEs are apparently inconsistent with previous literature that identified no difference, this previous literature did not normalize to a loading control as was performed here, which is a more accurate method (Gardner et al., 2020). Furthermore, this previous work did not evaluate the extracellular acid‐SMase levels as we did here. However, of note, this previous work did provide evidence suggesting that decreased CDase activity was the cause for increased ceramide levels in cfHBEs compared to nHBEs. This mechanism would be consistent with our observation that sphingomyelin levels are also increased in cfHBEs.

To provide further physiological context to our work, we also determined within this study that LTB4‐transmigrated neutrophils secrete acid‐SMase. Neutrophils are present at high numbers in the CF lung, and thus this secretion of acid‐SMase is highly relevant to understanding the disease state of CF (Giacalone et al., 2020). These results, along with the existing literature, suggest that determining the mechanism of WT SMase‐mediated decreases in CFTR activity in HBEs is quite relevant.

This study has shown that accumulation of ceramides and/or dihydroceramides underly the proximal mechanism by which SMase reduces CFTR activity in airway cells. However, the next steps in the mechanism by which ceramide and/or dihydroceramide could be affecting CFTR‐mediated conductance still remain to be understood completely. There are four ways that lipids can affect a membrane protein's activity: (1) direct allosteric interaction, (2) changing surface localization, (3) changing signaling cascades, and (4) membrane mechanics (Figure 1) (Cottrill et al., 2020). Our prior experiments in *Xenopus* oocytes showed that WT SMase applied directly to an excised patch of membrane did not inhibit CFTR channels within the patch (Stauffer et al., 2017). This indicated that direct allosteric interaction and membrane mechanics affected by the loss of sphingomyelin and accumulation of ceramide and phosphocholine did not underly the inhibition of CFTR conductance. This theoretically will be true regardless of cell type. Further experimentation in *Xenopus* oocytes showed that WT SMase did not change the surface expression of CFTR, a phenomenon that we confirmed in nHBEs in this study, using surface biotinylation. Instead, we found in *Xenopus* oocytes that intracellular components were necessary to facilitate WT SMase‐mediated decreases in CFTR currents, indicating that a signaling mechanism was involved (Stauffer et al., 2017). It is likely that a signaling mechanism is involved in HBEs as well.

As we established within this study and previously (Cottrill et al., 2021; Stauffer et al., 2017), VX770 does not rescue WT SMase‐mediated decreases in CFTR currents or conductances in nHBEs. We also have shown previously in nHBEs that potentiation of CFTR currents by VX770 is greater when CFTR has reduced levels of PKA phosphorylation (Cui et al., 2019). Therefore, if WT SMase inhibited CFTR activity by decreasing PKA phosphorylation, we would expect that VX770 treatment would have recovered the CFTR‐mediated current and conductance, which was not the case. Furthermore, we and others previously showed that in *Xenopus* oocytes, WT SMase inhibits CFTR channels without the R‐domain, the location of the PKA phosphorylation sites (Ramu et al., 2007; Stauffer et al., 2017). A signaling mechanism unrelated to PKA‐mediated phosphorylation must be involved.

Both ceramide and dihydroceramide activate AMPK (Ji et al., 2010; Siddique et al., 2013), which inhibits CFTR by phosphorylating S737 and S768 in the R‐domain. We utilized the AMPK inhibitor BML‐275 to determine its involvement in the mechanism of action of SMase and found that WT SMase decreased CFTR‐mediated currents to a similar degree even with this inhibitor present. This indicates that AMPK is not involved. Ceramide is known to initiate many other signaling cascades, though, including those involving protein phosphatases 2A and 2C (PP2A (Dobrowsky et al., 1993), PP2C (Perry et al., 2012)). PP2A and PP2C inhibit CFTR by dephosphorylating the R‐domain's stimulatory phosphorylation sites, thus inactivating CFTR (Luo et al., 1998; Thelin et al., 2005; Travis et al., 1997). This is functionally the same as reduced PKA phosphorylation, so we can hypothesize based on the lack of VX770‐mediated recovery that PP2A and PP2C are not involved in the inhibitory mechanism. However, we again note that the effect of SMase in *Xenopus* oocytes did not require the R‐domain.

There are still alternative mechanisms to explore. Ceramide inhibits certain protein kinase C isoforms (Bourbon et al., 2001). PKC has differential effects on CFTR, depending on which site it phosphorylates (Chappe et al., 2004). In Calu‐3 cells, the PKC activator phorbol 12‐myristate 13‐acetate did not recover the WT SMase‐mediated decrease in cAMP‐activated transepithelial anion currents (Ito et al., 2004), although we have shown previously that WT SMase does not affect CFTR currents in the same manner in Calu‐3s as in nHBEs (Cottrill et al., 2021). Future work should activate protein kinase C to determine if this enzyme is involved in WT SMase‐mediated inhibition of CFTR in nHBEs. Dihydroceramides are also known to decrease ATP levels (Siddique et al., 2013; Zheng et al., 2006), which could decrease CFTR‐mediated conductance since ATP is required for CFTR gating (Berger et al., 1991). Thus, along with the remainder of the future experiments suggested in this paper, future work should evaluate ATP levels to determine if these enzymes are involved in WT SMase‐mediated inhibition of CFTR in nHBEs.

### 4.1 Limitations

A limitation of this study is that the effects of ceranib‐1 and BML‐275 were only evaluated in regard to transepithelial currents. Future work should re‐evaluate these treatments in regard to their effects on apical and basolateral conductance. Furthermore, this study is limited in that only a single biological replicate was used to determine the imbalance of ceramides and dihydroceramides in CF airway cells as compared to non‐CF controls. However, we are sufficiently confident in the results presented here because similar trends were found in cfHBEs and cfHNEs as compared to nHBEs and nHNEs, all from different donors, and since previous literature has reported similar findings. This study is also limited in that only a single neutrophil donor was used to evaluate the protein expression of acid‐SMase in neutrophils. Future work should evaluate acid‐SMase expression in neutrophils from multiple donors.

## 5 CONCLUSIONS

We have shown that the bacterial virulence factor SMase, applied to airway epithelial cells, causes a reduction of CFTR current in nHAEs, and that inhibiting CDase with ceranib‐1 works through the same mechanism. Ceranib‐1 only increased total cellular dihydroceramides, which WT SMase also increased, but a localized effect on ceramides cannot be ruled out. WT SMase did not decrease CFTR currents by reducing total cellular CFTR abundance, CFTR surface expression, or by activating AMPK. More work is necessary to determine the complete molecular mechanism by which WT SMase decreases CFTR activity. This work is relevant, as we have found here that sphingomyelins, ceramides, dihydroceramides, and secreted acid‐SMase protein are increased in CF cells relative to non‐CF cells. Transmigrated neutrophils also were found to secrete acid‐SMase. Importantly, 48 h treatment with VX445 + VX661 as well as 48 h treatment with VX445 + VX661 + VX770 decreased all ceramides and some sphingomyelins, suggesting that misfolded mutant CFTR may play a key role in establishing these imbalances. However, these treatments also further increased a dihydroceramide, which could be of clinical importance and should be evaluated further.

## CONFLICT OF INTEREST

The authors have no conflict of interest to report.

## AUTHOR CONTRIBUTIONS

KAC performed experiments and wrote the manuscript. VDG and CM performed experiments involving transmigration of neutrophils. RJB provided bronchial epithelial cells. MK provided bronchial and nasal epithelial cells for study and expertise on sphingolipids. RT provided funding as well as expertise on CF, neutrophils, and transmigration. NAM provided funding as well as expertise on CF, CFTR, Ussing chamber, and bronchial epithelial cells.

## Supporting information



Figure S1
Figure S2

## Data Availability

Data are available upon request from N.A.M. (namccar@emory.edu).
