# Supplementary material for: Mechanistic analysis and significance of sphingomyelinase‐mediated decreases in transepithelial CFTR currents in nHBEs
Source: Physiol Rep. 2021 Sep 13;9(17):e15023. doi: 10.14814/phy2.15023 (PMC8436056; doi:10.14814/phy2.15023)
Supplement: Supplementary file 1 — Figure S1 Figure S2 [file PHY2-9-e15023-s001.docx]

**Supplemental Figures**


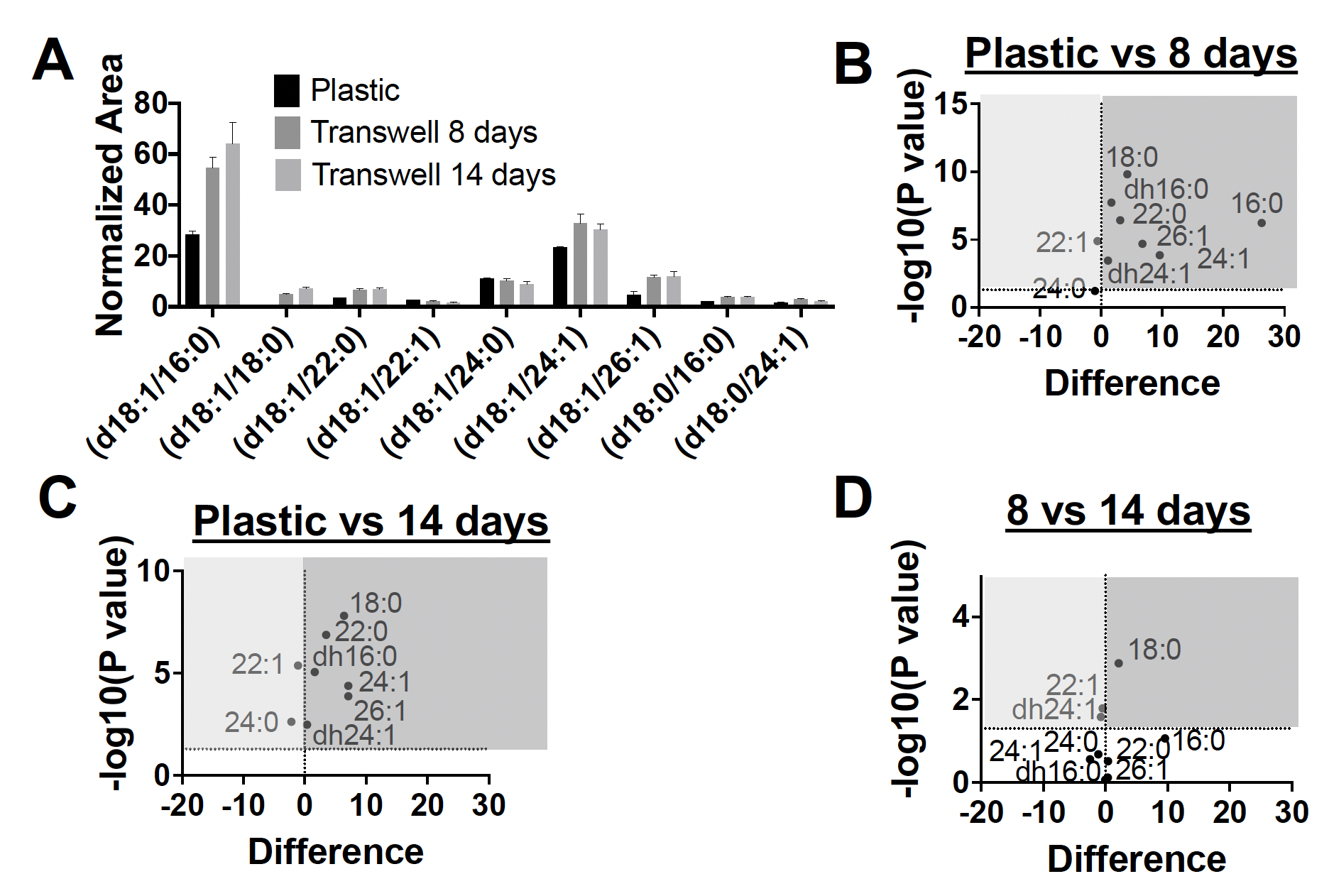


**Figure S1.** Lipidomics analysis was performed to evaluate ceramides and dihydroceramides on nHBEs grown either on T25 flasks (black, n=6), on Transwells for 8 days (dark grey, n=4), or on Transwells for 14 days (light grey, n=4). **(A)** A bar graph of normalized areas for each sphingolipid of interest is shown. **(B-D)** Data were analyzed by multiple t tests as described in Methods, and volcano plots were created. Lipids in the dark grey box are increased in the comparative treatment, while lipids in the light grey box are decreased. **(B)** All dihydroceramides and most ceramides increased when transitioning the cells from a T25 flask to a Transwell for 8 days. The exceptions were one decreased ceramide, and one ceramide that was unchanged. **(C)** By 14 days on the Transwell, this unchanged ceramide was found to be significantly decreased as compared to the T25 cells. **(D)** Also, by 14 days, one ceramide had further increased, one ceramide had further decreased, and dihydroceramide had decreased as compared to cells that were only on the Transwells for 8 days. This indicates that time on the Transwell affects the sphingolipid profile. This must be taken into consideration during future comparative experiments.

**Figure S2.** Western blot and densitometry analyses indicate imbalances in acid-SMase protein expression and secretion in cfHBEs as compared to nHBEs. **(A-B)** Cell lysate of cfHBEs had approximately 52% more protein than nHBEs. When correcting for this loading control, cfHBE cell lysate still had 22% more acid-SMase protein expression than nHBEs. **(C-D)** Basolateral medium of cfHBEs had approximately 44% less protein than nHBEs (100%-56%). When correcting for this loading control, basolateral medium from cfHBEs still had 11% more acid-SMase protein expression than nHBEs.
